# Nomogram for predicting survival in patients with advanced hepatocellular carcinoma treated with PD-1 inhibitors: incorporating pre-treatment and post-treatment clinical parameters

**DOI:** 10.1186/s12885-023-11064-1

**Published:** 2023-06-16

**Authors:** Guhe Jia, Lupeng Qiu, Hongye Zheng, Boyu Qin, Zhuoya Sun, Yangyang Shao, Zizhong Yang, Jiakang Shao, Yuxin Zhou, Shunchang Jiao

**Affiliations:** 1 School of Medicine, Nankai University, Tianjin, 300071 China; 2 Department of Medical Oncology, Chinese People’s Liberation Army General Hospital, Beijing, 100853 China; 3 Medical School of Chinese People’s Liberation Army, Chinese People’s Liberation Army General Hospital, Beijing, 100853 China; 4 Beijing DCTY ® Biotech CO., LTD, Beijing, 102200 China

**Keywords:** Advanced hepatocellular carcinoma (aHCC), PD-1 inhibitors, Neutrophil-to-lymphocyte ratio (NLR), Prognosis

## Abstract

**Background:**

Immunotherapy has transformed cancer treatment patterns for advanced hepatocellular carcinoma (aHCC) in recent years. Therefore, the identification of predictive biomarkers has important clinical implications.

**Methods:**

We collected medical records from 117 aHCC patients treated with anti-PD-1 antibody. Kaplan-Meier analysis and Cox proportional hazard regression were used to evaluate the association between peripheral blood biomarkers and overall survival (OS) and progression-free survival (PFS). Finally, the prognostic nomogram was constructed.

**Results:**

The mPFS and mOS were 7.0 months and 18.7 months, respectively. According to Kaplan-Meier analysis and Cox regression analysis, we regarded the treatment regimen (*p* = 0.020), hemoglobin (Hb) at 6-week (*p* = 0.042), neutrophil-to-lymphocyte ratio (NLR) at 6-week (*p* < 0.001), system immune inflammation index (SII) at 6-week (*p* = 0.125) as predictors of PFS, and alpha fetoprotein (AFP) (*p* = 0.035), platelet-to-lymphocyte ratio (PLR) (*p* = 0.012), Hb at 6-week (*p* = 0.010) and NLR at 6-week (*p* = 0.020) as predictors of OS. Furthermore, the results suggest that the OS and PFS nomogram model were in agreement with actual observations.

**Conclusion:**

Biomarkers in peripheral blood can predict the prognosis of patients with aHCC treated with anti-PD-1 antibody. The development of nomogram models can help us to screen potential patients who can benefit from immunotherapy.

## Introduction

Globally, the incidence of hepatocellular carcinoma (HCC) is the fifth-highest and second in mortality among malignant tumors with poor survival rate [1]. Among the five most common malignancies in the world, HCC is the only cancer with incidence and mortality increasing year by year [2]. Etiologically, hepatitis B/C virus infection and alcohol consumption are responsible for the deaths of more than 80% of patients with HCC. The incidence of HCC in China is high, and most cases are associated with chronic hepatitis B virus (HBV) infection [3].

For early-stage HCC patients, surgical resection, radiofrequency ablation, and liver transplantation are thought to be the curative treatment options [4]. But even with radical resection, 60–70% of patients still have a high risk of recurrence or metastasis within 5 years. 50% of HCC patients are already advanced at the initial diagnosis, and systemic therapy is the only viable option for them [5]. Sorafenib and lenvatinib have been approved as first-line targeted therapy for advanced HCC (aHCC), ushering in a new era of systemic therapy. However, after more than 10 years of subsequent studies, targeted therapy remains the only available systemic therapy for aHCC patients, while the median overall survival (OS) of sorafenib monotherapy as a first-line treatment of aHCC is 10.7 months [6]. In addition, the low target effects and substantial side effects, including fatigue, weight loss, diarrhea and rash, limited the utility of these drugs [7]. Obviously, molecular targeted therapy is not enough for aHCC patients.

Currently, immune checkpoint inhibitor (ICI) has been emerging as a promising treatment option for patients with aHCC [8]. In the CheckMate-040 trial, nivolumab demonstrated good efficacy and safety in second line treatment for aHCC [9]. As a result, nivolumab has been approved by the US Food and Drug Administration (FDA) for the treatment of aHCC [10]. In addition, sintilimab and tislelizumab have also shown good efficacy and safety in clinical trials [11, 12]. However, these anti-PD-1 antibody can lead to high treatment costs and immune-related adverse effects, so there is an urgent need to identify the patients who are most likely to benefit from anti-PD-1 antibody [13, 14].

PD-L1 expression is an FDA-approved companion biomarker of anti-PD-1 antibody response in lung, bladder, head and neck cancers [15]. But there are no well-established or validated biomarkers for HCC. In addition, the efficacy of PD-1 inhibitors varied widely between individuals, and the responses to anti-PD-1 therapy could be observed regardless of the baseline PD-L1 expression [16]. Therefore, PD-L1 expression cannot be used as a reference indicator for predicting the effect of immunotherapy, justifying the necessity of finding other predictive biomarkers to identify patients who may benefit from anti-PD-1 therapy. Some tumor tissue biomarkers, such as tumor mutational burden (TMB), mismatch repair protein (MMR), or microsatellite instability (MSI) are FDA-approved for anti-PD-1 in several advanced solid tumors. However, response biomarkers, which can only be detected by genetic testing or immunohistochemistry, may not be feasible due to the non-routine testing and high cost [14, 17].

A large number of epidemiological studies have found that tumors usually occur in sites of chronic inflammation, including colorectal cancer, lung cancer, breast cancer, etc. [18]. This infiltration of inflammatory cells can be frequently observed and cytokines are unregulated in tumors, so the relationship between tumor growth and inflammation is well-recognized [19]. In China, HBV infection plays an important etiological role in the development, since the body will generate a chronic inflammatory state under prolonged stimulation of HBV infection. In this context, it assists in the occurrence, development and metastasis of HCC [20]. Therefore, predicting HCC prognosis with inflammatory biomarkers appears to be a promising diagnostic approach.

Inflammatory factors have been reported to be significantly associated with clinical outcomes in HCC patients, revealing the predictive value of systemic inflammatory markers in HCC prognosis [21]. Furthermore, the nutritional status of patients is also related to cellular and humoral immunity, phagocytic activity and other defense system functions, which suggests that nutritional status may be beneficial to the prediction of prognosis [22].

This article aims to evaluate the comprehensive prognostic value of peripheral blood biomarkers in patients with aHCC undergoing immunotherapy. Finally, the progression-free survival (PFS) and OS nomograms were developed based on the results provided.

## Materials and methods

### Study design and participants

A total of 165 aHCC patients at Chinese People’s Liberation Army General Hospital who received continuous treatment with PD-1 inhibitors from January 1, 2016 to March 1, 2022 were reviewed. The inclusion criteria were: 1) clinical symptoms, imaging features and serological molecular markers are consistent with the diagnostic criteria of aHCC; 2) patients have received at least two courses of first-line anti-PD-1 treatment. The exclusion criteria were: 1) incomplete hematological data; 2) lack of follow-up. According to these criteria, 117 patients were enrolled in the final analysis. All patient data for the retrospective study was accessed in compliance with relevant data protection and privacy regulations. The collected tumor characteristics of patients included: age, gender, body mass index (BMI), tobacco and alcohol status, Eastern Cooperative Oncology Group performance status (ECOG PS), TNM stage, Child-Pugh stage, Barcelona Clinic Liver Cancer (BCLC) stage, hepatitis B infection, liver cirrhosis, extrahepatic metastasis, alpha fetoprotein (AFP), prior surgery, prior locoregional therapy and treatment regimen. This study was conducted in accordance with the Declaration of Helsinki, and approved by the Ethics Committee of Chinese PLA General Hospital (S2022-313-02). All methods were performed in accordance with relevant guidelines and regulations.

### Assessment of peripheral blood biomarkers

Hematological parameters were recorded for the period beginning 2 weeks before the first cycle of anti-PD-1 therapy up to 6 weeks after the start of therapy and included: hemoglobin (Hb), neutrophil-to-lymphocyte ratio (NLR) [23], platelet-to-lymphocyte ratio (PLR), lymphocyte-to-monocyte ratio (LMR) [24], system immune inflammation index (SII), aspartate Ratio of acid aminotransferase to alanine aminotransferase (AST/ALT), albumin (ALB), total bilirubin (TBIL), and lactate dehydrogenase (LDH). The above data was extracted from electronic medical records.

### Follow-up

Tumor assessment was performed at baseline and then at the discretion of the therapist on a regular basis. The response was evaluated by the researchers according to the solid tumor response assessment criteria (iRECIST) 1.1 of the researchers. The follow-up ended on July 13, 2022. Objective remission rate (ORR) was defined as the proportion of optimal remission patients with complete remission (CR) or partial remission (PR), and disease control rate (DCR) was defined as CR, PR or stable proportional disease (SD). Total survival (OS) is calculated from the first cycle of immunotherapy to the time of death or the duration of the last follow-up, while progression-free survival (PFS) is from the first cycle of immunotherapy to the duration of imaging recorded disease progression or last follow-up.

### Statistical analysis

We first evaluated the biomarkers in patients with aHCC. The peripheral blood biomarkers of the patients were regarded as continuous variables. X-tile software was used to determine the best cutoff value of hematological parameters. Chi-square test or Fisher exact test was used to determine the univariate association between classified variables and treatment response. Kaplan-Meier method and logarithmic rank test were used for survival analysis. Cox proportional hazard regression analysis was performed to determine potential indicators related to PFS and OS. The factors with P value less than 0.05in univariate Cox regression analysis were further included in multivariate analysis to determine the factors independently related to survival. The R package “rma” was used to build the nomogram model for predicting. The calibration curve and the area under the curve (AUC) were used to evaluate the correlation between the actual results and the predicted probability. R software and SPSS 26.0 were used for statistical analysis. All p values were two-sided, and a P value less than 0.05 was considered to be statistically significant.

## Results

### Baseline characteristics

A total of 117 patients with aHCC who received PD-1 inhibitor were included in the study. The patients baseline characteristics are shown in Table 1. 20.5% of patients (n = 24) were elderly people (> 65) (median of 58, IQR [51–65]), and the patients were predominantly males (n = 106, 90.6%). 42.7% of patients had a BMI greater than 24, and a large majority (n = 108, 92.3%) of patients had a pretreatment ECOG performance status of 0 or 1. 45.3% of patients (n = 53) had TNM stage III, and 54.7% (n = 64) had stage IV. 28.2% of patients (n = 33) were diagnosed with BCLC stage B, and 71.8% (n = 84) with BCLC stage C. 59.0% of patients (n = 69) had Child Pugh class A liver function, and 41.0% (n = 48) had class B. .79.5% of patients (n = 93) had viral hepatitis, and 55.6% (n = 65) had liver cirrhosis. Moreover, 44.4% of patients (n = 52) had extrahepatic metastasis, and 68.4% of patients (n = 80) had an AFP level of more than 400 (median of 31, IQR [5–2586]). Almost half of the patients (n = 52, 44.4%) underwent liver cancer resection, and a total of 58.1% of patients (n = 68) received chemotherapy, radiofrequency ablation, transcatheter arterial chemoembolization (TACE) or radiotherapy. As for treatment regimen, 20.5% of patients (n = 24) received ICI monotherapy, and 79.5% of patients (n = 93) received anti-PD-1-based combination therapy. Among the 93 patients treated with combination immunotherapy, most patients (n = 88, 94.6%) received immunotherapy combined with targeted therapy including lenvatinib (n = 38, 40.9%), anlotinib (n = 20, 21.5%), apatinib (n = 9, 9.7%), sorafenib (n = 3, 3.2%) and bevacizumab (n = 18, 19.4%), whereas 5.4% of patients (n = 5) had immunotherapy combined with chemotherapy.

**Table 1:** Baseline characteristics

Characteristics	N (%)
Number of patients	117 (100)
Age	
≤65	93 (79.5)
>65	24 (20.5)
Sex	
Male	106 (90.6)
Female	11 (9.4)
BMI (kg/m^2^)	
≤24	67 (57.3)
>24	50 (42.7)
ECOG PS	
0	88 (75.2)
1	20 (17.1)
≥2	9 (7.7)
TNM stage	
III	53 (45.3)
IV	64 (54.7)
Child-Pugh stage	
A	69 (59.0)
B	44 (37.6)
C	4 (3.4)
BCLC stage	
B	33 (28.2)
C	84 (71.8)
Hepatitis B infection	
No	24 (20.5)
Yes	93 (79.5)
Liver cirrhosis	
No	52 (44.4)
Yes	65 (55.6)
Extrahepatic metastasis	
No	63 (53.8)
Yes	54 (46.2)
AFP (ng/mL)	
≤400	80 (68.4)
>400	37 (31.6)
Prior surgery	
No	65 (55.6)
Yes	52 (44.4)
Prior locoregional therapy	
No	49 (41.9)
Yes	68 (58.1)
Macro vascular invasion	
No	49 (41.9)
Yes	68 (58.1)
Treatment regimen	
Monotherapy	24 (20.5)
Combination therapy	93 (79.5)

ECOG PS: Eastern Cooperative Oncology Group performance status; BCLC: Barcelona Clinic Liver Cancer; AFP: alpha fetoprotein

### The treatment response of aHCC patients treated with anti-PD-1 therapy

**Fig. 1:**
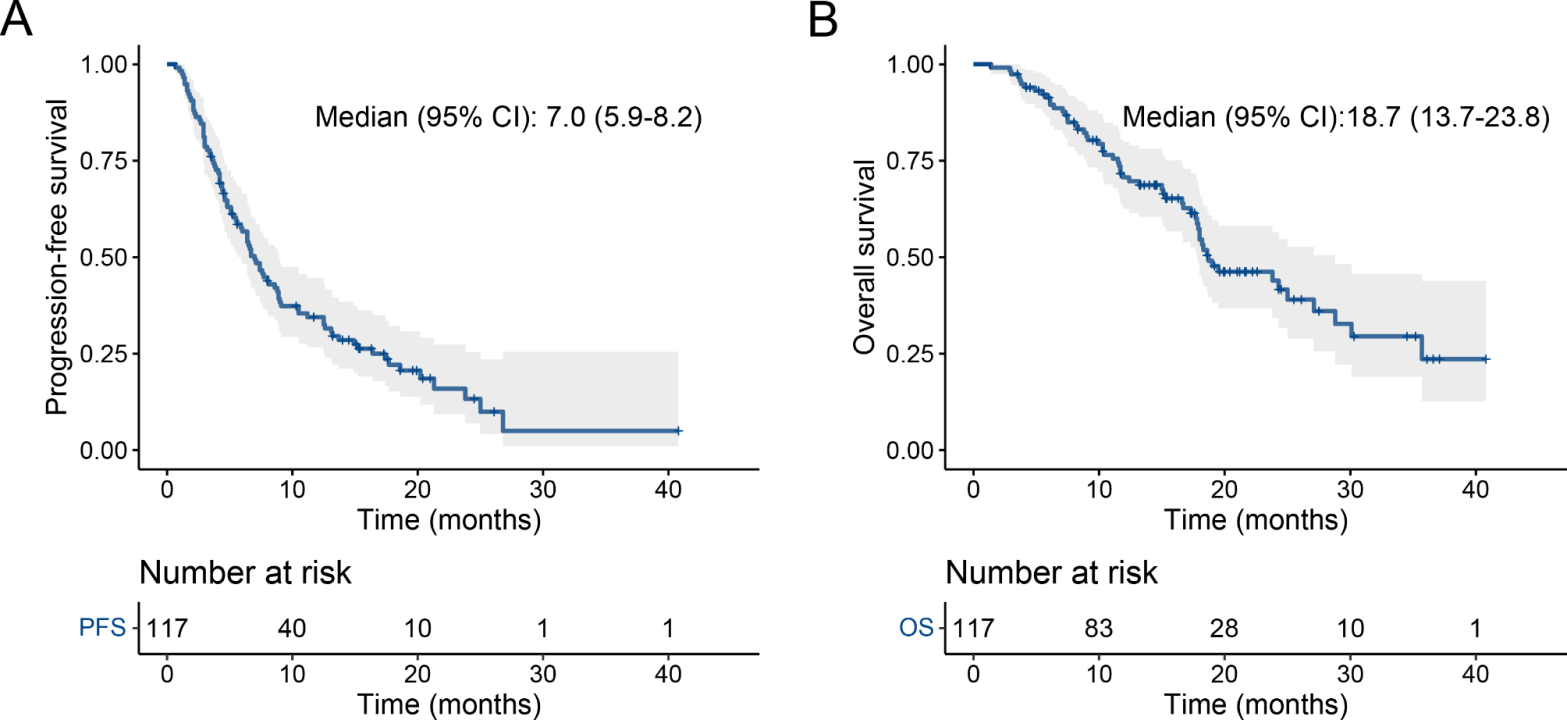
Overall population survival curves. PFS **(A)**, OS **(B)**

As of the data cutoff on July 13, 2022, the median duration of follow-up was 15.1 months. There was 1 (0.8%) case with complete response, 14 (11.9%) cases with partial response, 76 (64.9%) cases with stable disease, and 26 (22.2%) cases with progressive disease. Therefore, the ORR was 12.7%, and the DCR was 77.6%. 58 patients (49.5%) had died, and 26 patients (22.2%) remained at the progression-free stage. The median PFS were 7.0 months (95% CI: 5.9 to 8.2 months; Fig. 1A), while the median OS were 18.7 months (95%CI: 13.7 to 23.8 months; Fig. 1B).

### Clinical prediction model for PFS in patients with aHCC

**Fig. 2:**
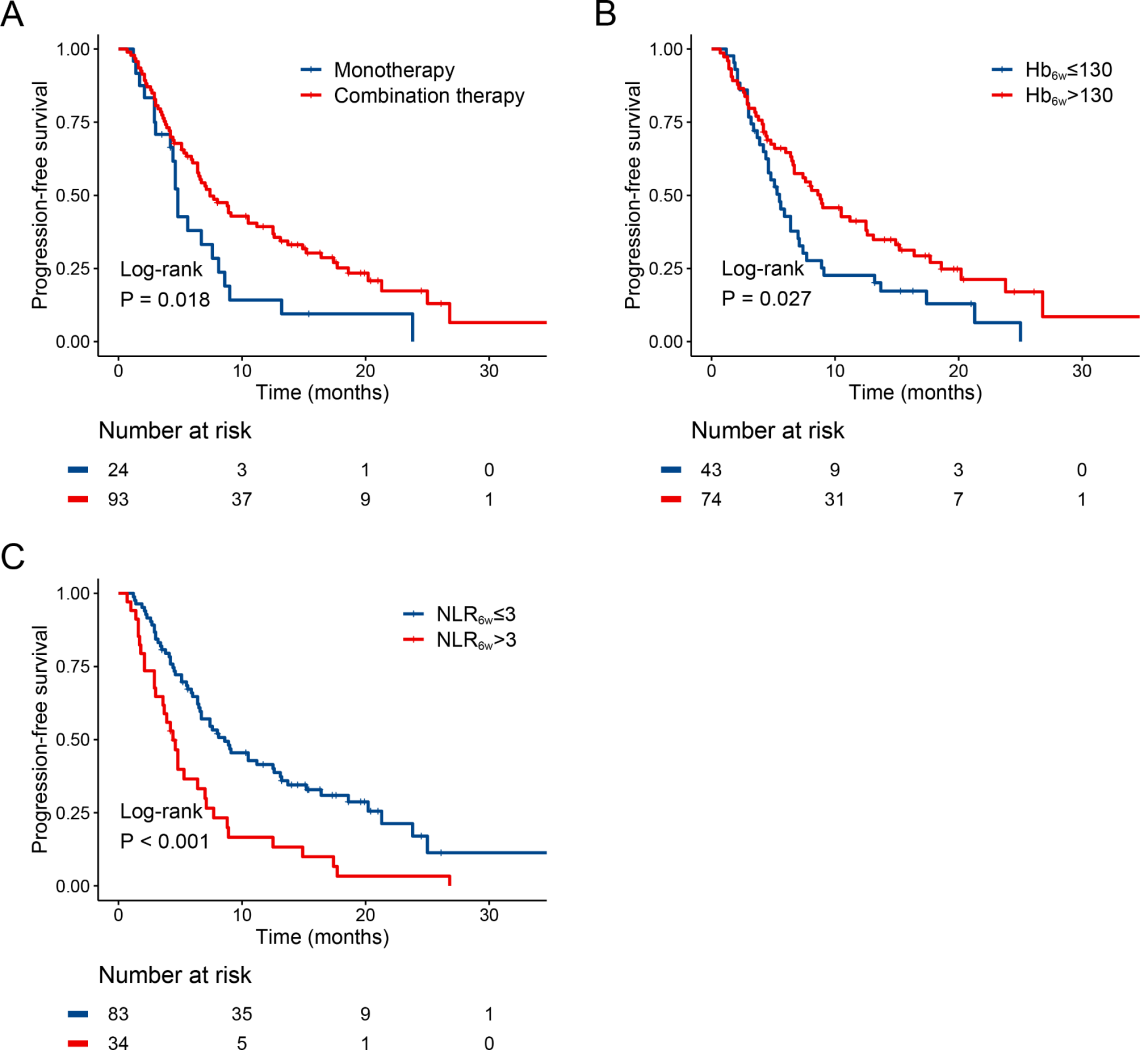
PFS curve. treatment regimen **(A)**, Hb at 6-week **(B)**, and NLR at 6-week **(C)**. HR: hazard ratio; CI: confidence interval; HB: hemoglobin; NLR: neutrophil-to-lymphocyte ratio

The univariate Cox regression analyses showed that treatment regimen (*p* = 0.020), Hb at 6-week (*p* = 0.042), NLR at 6-week (*p* < 0.001), SII at 6-week (*p* = 0.125) were significantly associated with a prolonged PFS (Table 2). Those results from the prior univariable analysis were entered into multivariate analysis, which showed PFS was related to treatment regimen (HR: 0.58, 95% CI: 0.34–0.91; *p* = 0.020), Hb at 6-week (HR: 0.64, 95% CI: 0.42–0.98; *p* = 0.042), NLR at 6-week (HR: 2.19, 95% CI: 1.41–3.41; *p* < 0.001) (Table 2). Figure 2 shows the Kaplan–Meier curves of PFS for patients with aHCC receiving anti-PD-1 inhibitor. The monotherapy group had poorer mPFS compared to the combination therapy group (mPFS, 4.8 months, 95% CI: 4.4–5.2 vs. 7.4 months, 95% CI: 5.3–9.5, *p* = 0.018) (Fig. 2A). Compared to the Hb at 6-week > 130, patients with Hb at 6-week ≤ 130 had a significantly shorter mPFS (mPFS, 8.8 months, 95% CI: 6.1–11.5 vs. 5.5 months, 95% CI: 4.2–6.8, *p* = 0.027) (Fig. 2B). Moreover, the mPFS was shorter in patients with NLR at 6-week > 3 than those with NLR at 6-week ≤ 3 (mPFS 11.2 months, 95% CI: 5.9–16.5 vs. 5.3 months, 95% CI: 3.5–7.1, *p* < 0.001) (Fig. 2C).

**Table 2:** Results of univariate and multivariate Cox regression analysis for PFS

Variable	Category	Univariate analysis			Multivariate analysis		
		HR (95% CI)	P-value		HR (95% CI)	P-value	
**Clinical characteristics**							
Age	> 65 vs. ≤ 65	1.30 (0.76–2.23)	0.345				
Sex	female vs. male	1.15 (0.57–2.29)	0.701				
ECOG PS	≥ 2 vs. 0–1	1.20 (0.55–2.61)	0.655				
TNM stage	III vs. IV	1.44 (0.95–2.20)	0.087				
Child-Pugh stage	B + C vs. A	1.12 (0.74–1.71)	0.580				
BCLC stage	C vs. B	1.45 (0.89–2.37)	0.968				
Hepatitis B infection	yes vs. no	0.87 (0.52–1.46)	0.589				
Liver cirrhosis	yes vs. no	1.04 (0.69–1.57)	0.865				
Extrahepatic metastasis	yes vs. no	1.37 (0.90–2.07)	0.140				
AFP, ng/mL	> 400 vs. ≤ 400	1.41 (0.90–2.21)	0.132				
Prior surgery	yes vs. no	0.77 (0.51–1.17)	0.092				
Prior locoregional therapy	yes vs. no	1.02 (0.67–1.55)	0.924				
Macro vascular invasion	yes vs. no	1.08 (0.71–1.64)	0.718				
Treatment regimen	combination therapy vs. monotherapy	0.55 (0.34–0.91)	0.020		0.58 (0.35–0.95)	0.032	
**Pre-treatment hematologic markers**							
Hb, g/L	> 130 vs. ≤ 130	0.88 (0.58–1.35)	0.567				
NLR	> 3.0 vs. ≤ 3.0	1.26 (0.81–1.97)	0.312				
PLR	> 131 vs. ≤ 131	1.19 (0.77–1.84)	0.426				
LMR	> 3.3 vs. ≤ 3.3	0.90 (0.59–1.37)	0.626				
SII	> 509 vs. ≤ 509	1.16 (0.74–1.81)	0.528				
AST/ALT	> 1.3 vs. ≤ 1.3	1.15 (0.76–1.75)	0.505				
ALB, g/L	> 33 vs. ≤ 33	0.67 (0.44–1.02)	0.063				
TBIL, µmol/L	> 10 vs. ≤ 10	0.99 (0.64–1.54)	0.969				
LDH, U/L	> 184 vs. ≤ 184	1.16 (0.77–1.76)	0.476				
**Post-treatment hematologic markers**							
Hb at 6-week, g/L	> 130 vs. ≤ 130	0.62 (0.41–0.95)	0.028		0.64 (0.42–0.98)	0.042	
NLR at 6-week	> 3.0 vs. ≤ 3.0	2.23 (1.44–3.46)	< 0.001		2.19 (1.41–3.41)	< 0.001	
PLR at 6-week	> 131 vs. ≤ 131	1.26 (0.82–1.92)	0.287				
LMR at 6-week	> 3.3 vs. ≤ 3.3	0.92 (0.61–1.40)	0.713				
SII at 6-week	> 509 vs. ≤ 509	1.78 (1.15–2.77)	0.010		1.48 (0.90–2.45)	0.125	
AST/ALT at 6-week	> 1.3 vs. ≤ 1.3	1.40 (0.92–2.12)	0.113				
ALB at 6-week, g/L	> 33 vs. ≤ 33	0.70 (0.46–1.08)	0.103				
TBIL at 6-week, µmol/L	> 10 vs. ≤ 10	1.56 (0.98–2.48)	0.061				
LDH at 6-week, U/L	> 184 vs. ≤ 184	0.99 (0.65–1.52)	0.973				

HB: hemoglobin; NLR: neutrophil-to-lymphocyte ratio; PLR: platelet-to-lymphocyte ratio; LMR: lymphocyte-to-monocyte ratio; SII: system immune inflammation index, AST/ALT: aspartate Ratio of acid aminotransferase to alanine aminotransferase; ALB: albumin; TBIL: total bilirubin, LDH: lactate dehydrogenase

**Fig. 3:**
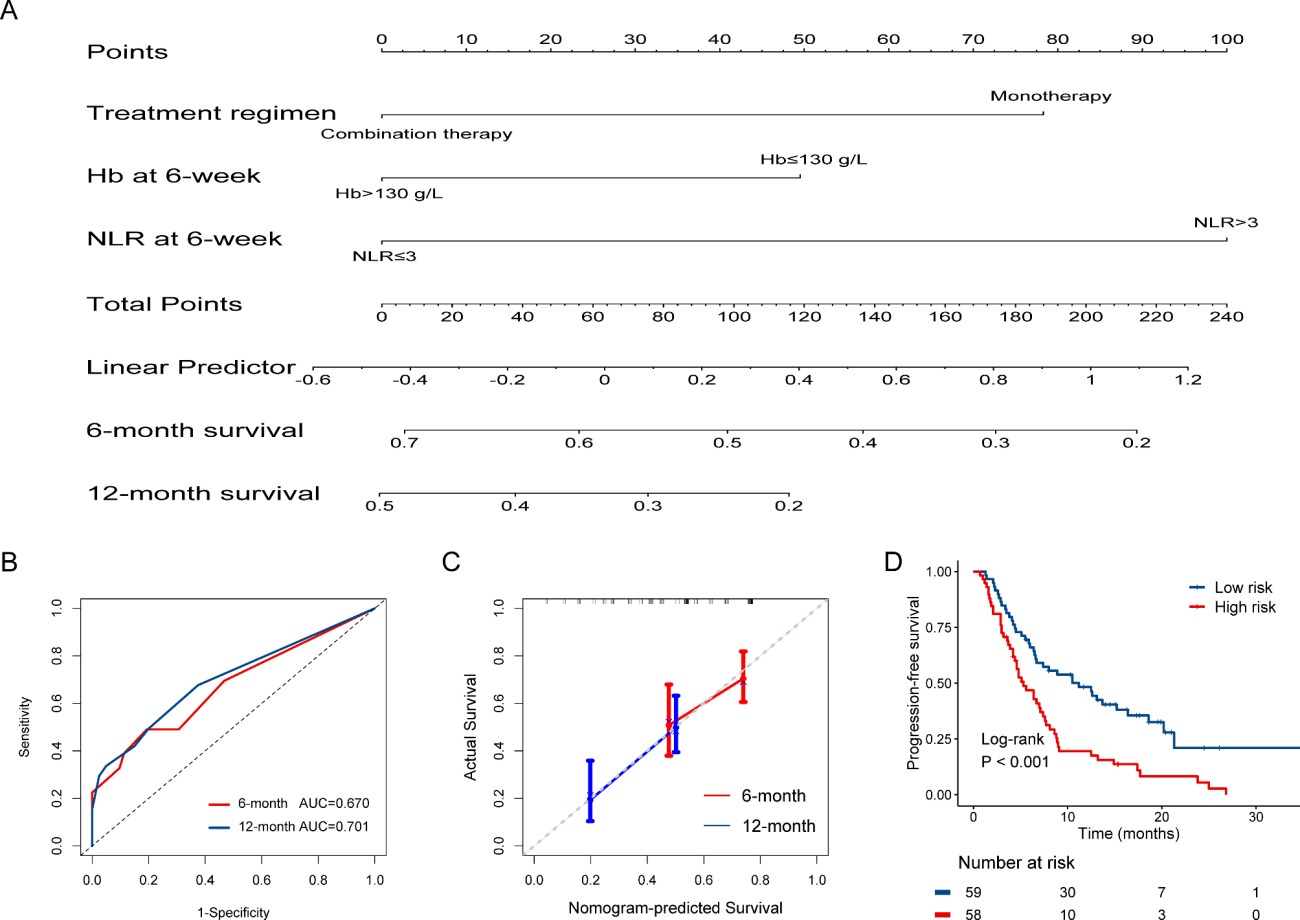
PFS prediction model. Drawing a line down to read the corresponding predicted probability **(A)**. ROC for the model **(B)**. The calibration curve for the nomogram to predict 6- and 12-month survival **(C)**. Kaplan-Meier curves using model predicted scores **(D)**. AUC: area under the curve; HR: hazard ratio; CI: confidence interval; PFS: progression-free survival; ROC: receiver operating characteristic curve

By establishing a nomogram that integrated the clinical factors and two peripheral blood biomarkers, a quantitative prognostic model was created. One value is matched for each factor, and then the factor values are added together to obtain the total score of the individual, which can help predict the PFS at 6- and 12- months (Fig. 3A). The results of receiver operating characteristic (ROC) analysis showed a 6-month AUC of 0.670 (95% CI: 0.527–0.769) and a 12-month AUC of 0.701 (95% CI: 0.611–0.790) (Fig. 3B). The 6- and 12-month calibration curves showed a good correlation between the actual and predicted outcomes (Fig. 3C). After calculating the risk score, 48 cases were classified as having a higher risk score and 49 cases were classified as having a lower risk score. Moreover, in contrast to the higher risk score, patients with lower risk score had a significantly longer PFS (mPFS, 5.3 months, 95% CI: 3.5–7.1 vs. 11.2 months, 95% CI: 5.9–16.5, *p* < 0.001; Fig. 3D).

**Fig. 4:**
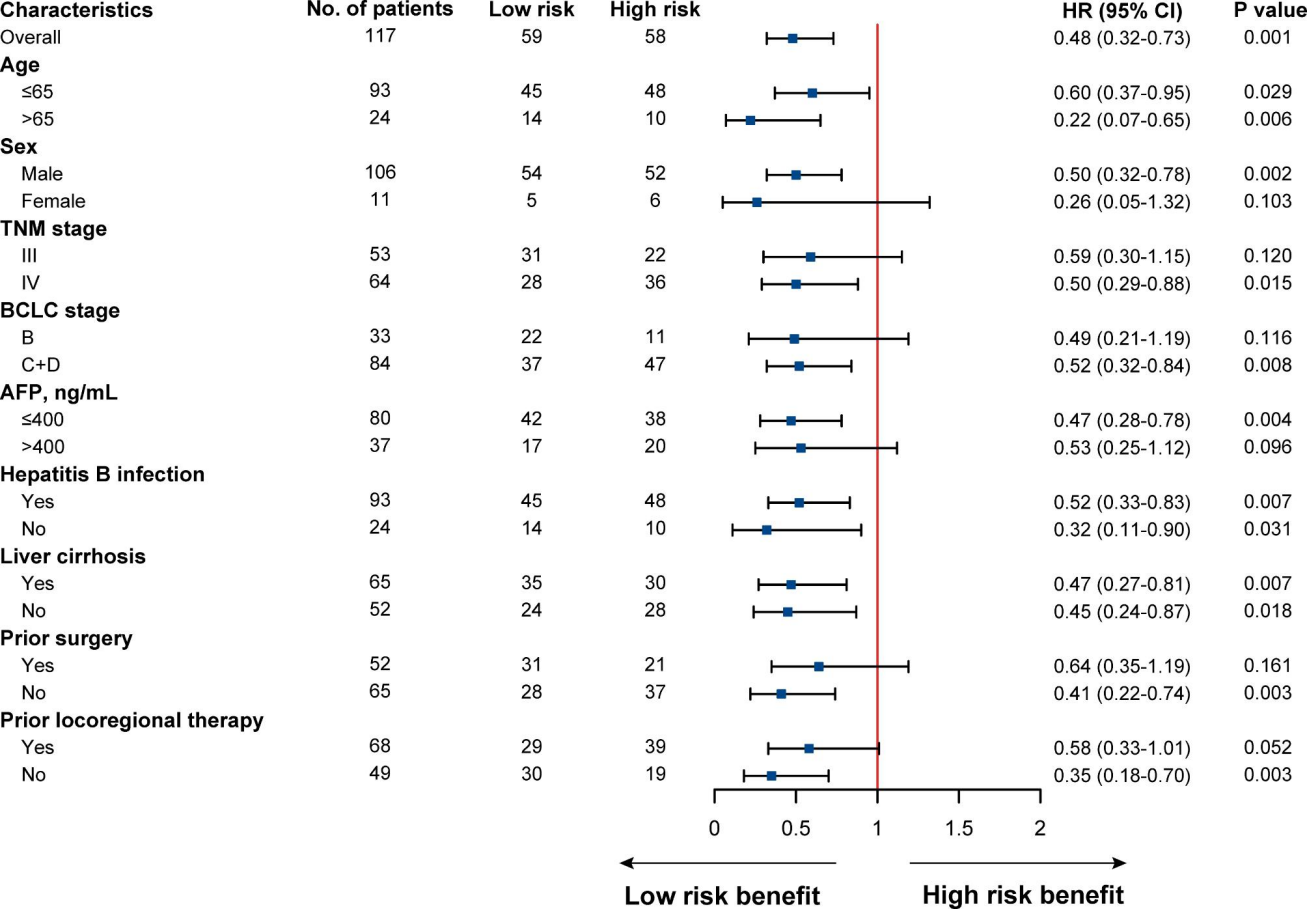
Forest plot of PFS

To further test the stability of our nomogram model, subgroup analyses were performed to describe the relationship between risk group and survival. The forest plot demonstrated that patients in the low-risk group tended to be associated with significantly longer PFS in most subgroups, including age, HBV infection, cirrhosis, and so on (Fig. 4).

### Clinical prediction model for OS in patients with aHCC

**Fig. 5:**
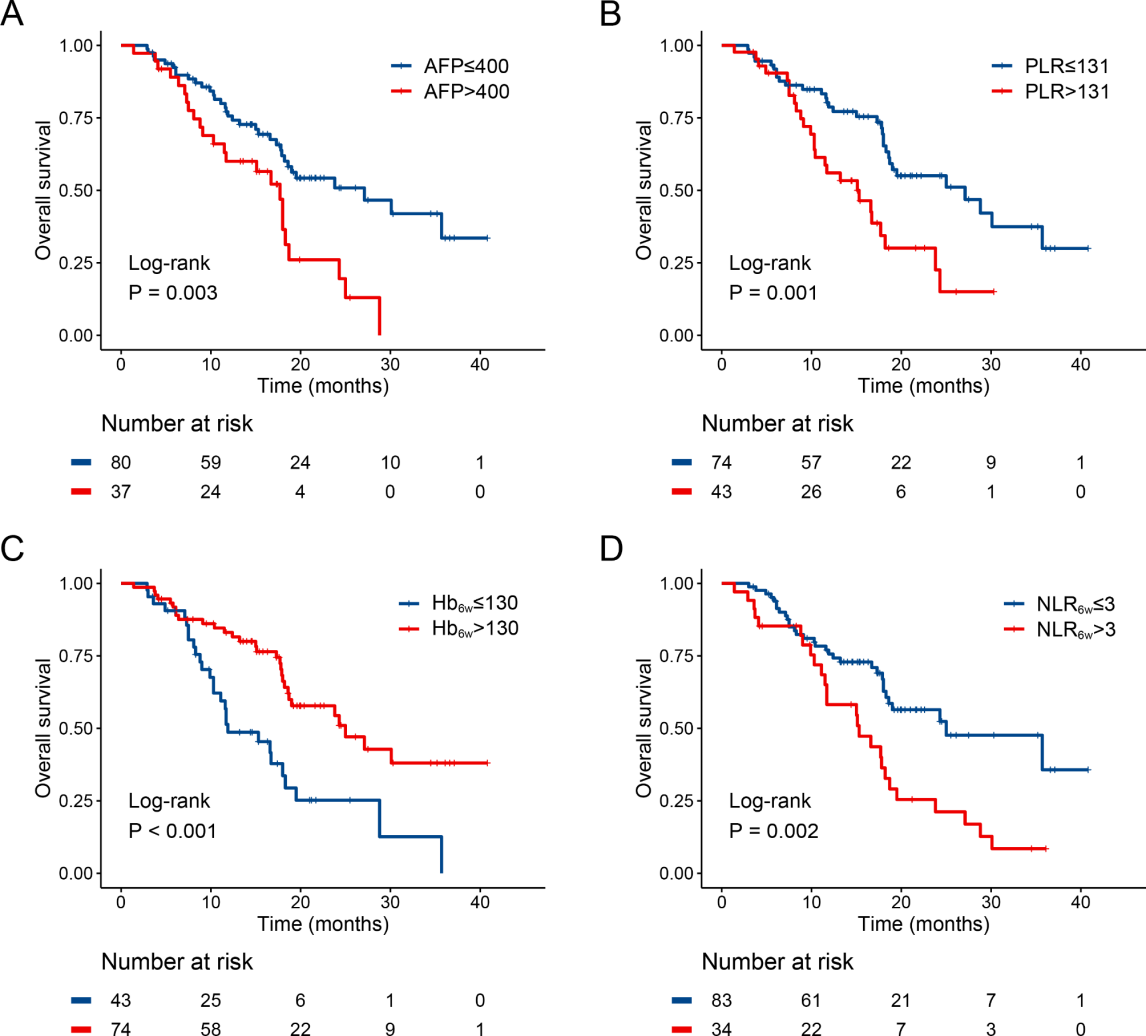
OS curve. AFP **(A)**, PLR **(B)**, Hb at 6-week **(C)** and NLR at 6-week **(D)**. HR: hazard ratio; CI: confidence interval; ECOG PS: Eastern Cooperative Oncology Group performance status; AFP: alpha fetoprotein; PLR: platelet-to-lymphocyte ratio; HB: hemoglobin; NLR: neutrophil-to-lymphocyte ratio

The univariate Cox regression analyses showed that AFP, NLR, PLR, SII, AST/ALT, Hb at 6-week, NLR at 6-week, PLR at 6-week, SII at 6-week, AST/ALT at 6-week were associated with prolonged OS (Table 3). The multifactor analysis showed that patients with decreased AFP (HR: 1.79, 95% CI: 1.04–3.08; *p* = 0.035), decreased PLR (HR: 2.09, 95% CI: 1.18–3.71; *p* = 0.012), increased Hb at 6-week (HR: 0.48, 95% CI: 0.27–0.84; *p* = 0.010) and decreased NLR at 6-week (HR: 1.88, 95% CI: 1.11–3.21; *p* = 0.020) had a longer OS (Table 3). Figure 5 shows the Kaplan–Meier curves of OS for patients with aHCC receiving an-ti-PD-1 inhibitor. Compared to the AFP > 400, patients with AFP ≤ 400 had a significantly longer mOS (mOS, 17.7 months, 95% CI: 15.0-20.4 vs. 27.1 months, 95% CI: 15.7–38.5, *p* = 0.003) (Fig. 5A). Patient with PLR > 131 had a significantly shorter mOS than patient with PLR ≤ 131 (mOS, 15.3 months, 95% CI: 9.3–21.3 vs. 27.1 months, 95% CI: 16.0-38.2, *p* = 0.001) (Fig. 5B). The patients with Hb at 6-week > 130 had a significantly longer mOS than the patients with Hb at 6-week ≤ 130 (mOS, 25.0 months, 95% CI 9.3–21.3 vs. 27.1 months, 95%CI 16.0-38.2, *p* < 0.001) (Fig. 5C). Moreover, in contrast to the patients with NLR at 6-week ≤ 3, the patients with NLR at 6-week > 3 had a significantly poorer mOS (mOS, 25 months, 95% CI: 12.5–37.5 vs. 15.3 months, 95% CI: 9.2–21.4, *p* = 0.002) (Fig. 5D).

**Table 3:** Results of univariate and multivariate Cox regression analysis for OS

Variable	Category	Univariate analysis			Multivariate analysis		
		HR (95% CI)	P-value		HR (95% CI)	P-value	
**Clinical characteristics**							
Age	> 65 vs. ≤ 65	1.49 (0.73–3.04)	0.274				
Sex	female vs. male	1.14 (0.51–2.52)	0.753				
ECOG PS	≥ 2 vs. 0–1	1.23 (0.53–2.88)	0.627				
TNM stage	III vs. IV	1.40 (0.81–2.41)	0.229				
Child-Pugh stage	B + C vs. A	1.52 (0.91–2.55)	0.112				
BCLC stage	C vs. B	0.99 (0.55–1.79)	0.968				
Hepatitis B infection	yes vs. no	1.07 (0.57–2.02)	0.837				
Liver cirrhosis	yes vs. no	1.45 (0.86–2.47)	0.167				
Extrahepatic metastasis	yes vs. no	1.31 (0.78–2.22)	0.307				
AFP, ng/mL	> 400 vs. ≤ 400	2.20 (1.29–3.77)	0.004		1.79 (1.04–3.08)	0.035	
Prior surgery	yes vs. no	0.64 (0.38–1.08)	0.092				
Prior locoregional therapy	yes vs. no	1.03 (0.61–1.73)	0.925				
Macro vascular invasion	yes vs. no	0.90 (0.53–1.52)	0.686				
Treatment regimen	combination therapy vs. monotherapy	0.61 (0.34–1.08)	0.091				
**Pre-treatment hematologic markers**							
Hb, g/L	> 130 vs. ≤ 130	0.61 (0.36–1.03)	0.065				
NLR	> 3.0 vs. ≤ 3.0	2.09 (1.22–3.58)	0.007		1.22 (0.61–2.45)	0.576	
PLR	> 131 vs. ≤ 131	2.32 (1.36–3.97)	0.002		2.09 (1.18–3.71)	0.012	
LMR	> 3.3 vs. ≤ 3.3	0.68 (0.40–1.12)	0.155				
SII	> 509 vs. ≤ 509	1.74 (1.01–2.99)	0.047		1.56 (0.90–2.72)	0.477	
AST/ALT	> 1.3 vs. ≤ 1.3	1.80 (1.07–3.03)	0.026		1.65 (0.91–2.99)	0.099	
ALB, g/L	> 33 vs. ≤ 33	0.65 (0.39–1.09)	0.105				
TBIL, µmol/L	> 10 vs. ≤ 10	1.75 (0.96–3.20)	0.069				
LDH, U/L	> 184 vs. ≤ 184	1.15 (0.69–1.94)	0.590				
**Post-treatment hematologic markers**							
Hb at 6-week, g/L	> 130 vs. ≤ 130	0.39 (0.23–0.66)	< 0.001		0.48 (0.27–0.84)	0.010	
NLR at 6-week	> 3.0 vs. ≤ 3.0	2.25 (1.34–3.78)	0.002		1.88 (1.11–3.21)	0.020	
PLR at 6-week	> 131 vs. ≤ 131	2.11 (1.25–3.56)	0.005		1.11 (0.56–2.21)	0.773	
LMR at 6-week	> 3.3 vs. ≤ 3.3	0.75 (0.44–1.28)	0.296				
SII at 6-week	> 509 vs. ≤ 509	2.00 (1.18–3.39)	0.010		1.59 (0.76–3.35)	0.220	
AST/ALT at 6-week	> 1.3 vs. ≤ 1.3	1.77 (1.05–2.99)	0.026		1.12 (0.62-2.00)	0.707	
ALB at 6-week, g/L	> 33 vs. ≤ 33	0.74 (0.44–1.25)	0.264				
TBIL at 6-week, µmol/L	> 10 vs. ≤ 10	1.60 (0.86–2.96)	0.139				
LDH at 6-week, U/L	> 184 vs. ≤ 184	1.23 (0.73–2.09)	0.440				

**Fig. 6:**
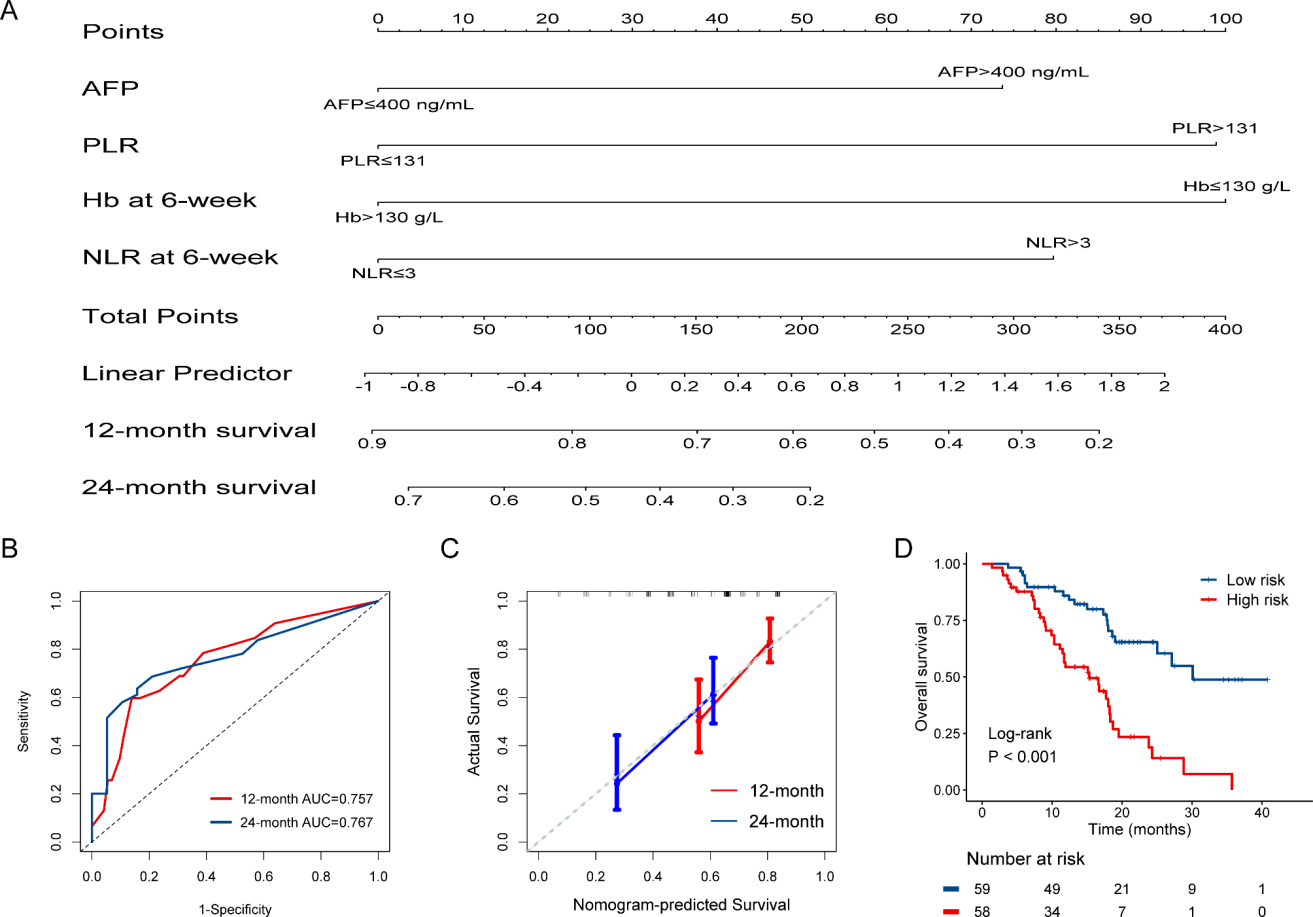
OS prediction model. Drawing a line down to read the corresponding predicted probability **(A)**. ROC for the model **(B)**. The calibration curve for the nomogram to predict 6- and 12-month survival **(C)**. Kaplan-Meier curves using model predicted scores **(D)**. AUC: area under the curve; HR: hazard ratio; CI: confidence interval; OS: overall survival; ROC: receiver operating characteristic curve

Similarly, a prognostic nomogram was created for OS based on the Cox proportional hazard regression model (Fig. 6A). The results of receiver operating characteristic analysis showed a 6-mouth AUC of 0.757 (95% CI: 0.655–0.858) and 12-mouth AUC of 0.767 (95% CI: 0.653–0.881) (Fig. 6B). The 6- and 12-month calibration curves showed a good correlation between the actual and predicted outcomes (Fig. 6C). After calculating the risk score, 58 cases were classified as having a higher risk score and 49 cases were classified as having a lower risk score. In comparison to the lower risk scores, patients with a higher risk score had a significantly poorer mOS (mOS, 15.3 months, 95% CI: 9.8–20.8 vs. 30.1 months, 95% CI: not reached, *p* < 0.001; Fig. 6D).

**Fig. 7:**
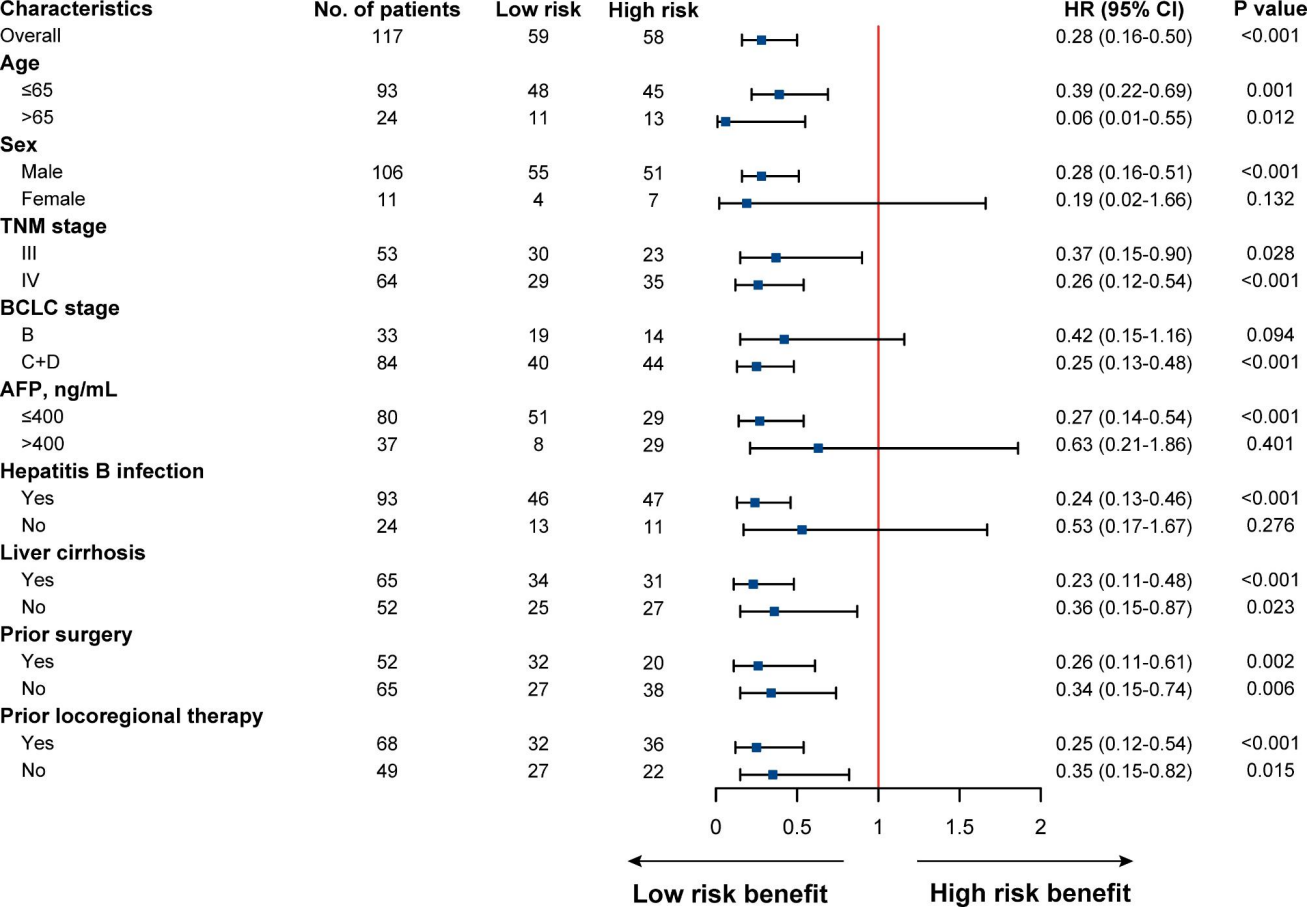
Forest plot of OS

Subsequently, subgroup analysis stratified by patients’ characteristics was conducted to further evaluate the predictive value of the OS nomogram. The forest plot indicated that patients with low-risk scores tended to be correlated with significantly longer OS in most subgroups, except for patients with female, BCLC stage B, higher AFP level, and non-B hepatitis (Fig. 7). Taken together, the nomogram model could serve as a stable and powerful tool for survival prediction, and can help to stratify aHCC patients who might benefit from immunotherapy.

## Discussion

It is worth mentioning that immunotherapy for aHCC has made breakthroughs in recent years, and anti-PD-1 antibodies play an important role in the treatment of aHCC patients. But due to the high cost of immune drugs and immune-related adverse reactions, some patients cannot get any survival benefits from these drugs. So, it is necessary to discover and develop the predictive biomarkers markers. We investigated the correlation between peripheral blood biomarkers and prognosis of immunotherapy based on PD-1 inhibitors in patients with aHCC before and after treatment.

As is well-established, inflammation plays a key role in the growth and spread of many kinds of tumors. High levels of peripheral neutrophils become the main mediators of tumor progression because they promote tumor cell growth and metastasis by inhibiting apoptosis and assisting angiogenesis [25]. In contrast, lymphocytes are involved in the regulation of cellular immunity, which is important for destroying tumor cells and associated micro metastases [26]. In this study, the mPFS were 7.0 months while the mOS were 18.7 months. Multivariate COX regression analysis showed that treatment regimen, Hb at 6-week and NLR at 6-week were independent prognostic factors for PFS. The AFP, PLR, Hb at 6-week and NLR at 6-week were independent factors of prognosis for OS. The Kaplan–Meier curves showed that patients with combination therapy, Hb at 6-week > 130, NLR at 6-week ≤ 3 had a significantly longer mPFS, and patients with AFP ≤ 400, PLR ≤ 131 group, Hb at 6-week > 130, NLR at 6-week > 3 had a significantly longer mOS.

These findings suggest that the above markers are potential prognostic markers in aHCC treated with anti-PD-1 antibodies. In this study, the related peripheral blood biomarkers were used to establish clinical predictive models to evaluate patients with aHCC more intuitively. We found the patients with lower risk scores had a significantly longer OS. To understand the effectiveness of the nomogram model, we also compared the association between risk score and patient prognosis. The results showed that the prediction performance of PFS and OS is moderate. To further validate our nomogram model, subgroup analyses were performed to describe the relationship between risk group and survival. The forest plot showed that patients in the low-risk group tended to be associated with significantly longer PFS and OS in most subgroups. These results suggest that the nomogram model could serve as a stable and powerful tool to stratify aHCC patients who might benefit from immunotherapy.

The results of the current study are consistent with those of previous studies. Hung et al [23] evaluated the effect of NLR on survival in 45 patients with aHCC who were treated with nivolumab. They reported that serum NLR (*p* = 0.025, 95% CI 1.10–3.80) was an independent risk factor for tumor progression in multivariate analysis. In addition, in a study of patients with aHCC treated with nivolumab monoclonal antibody, Sirish et al [24] concluded that NLR (*p* < 0.001, 95% CI: 1.05–1.15) after treatment was significantly associated with OS, and that OS was significantly longer in patients with NLR < 5 vs ≥ 5 before treatment (16 vs 5 months, *p* = 0.022) and after treatment (35 vs 5 months, *p* < 0.001). After treatment, PLR (*p* < 0.001) was closely related to survival rate. Recently, Yuji et al. [27] studied the relationship between survival outcome and baseline NLR in patients with aHCC treated with atezolizumab. The pre-treatment NLR value of patients with disease control was significantly lower than that of patients with disease progression (2.47 vs. 4.48 months, *p* = 0.013). Patients with NLR ≤ 3.21 had significantly better progression-free survival than patients with NLR > 3.21 (*p* < 0.001).

As far as we know, this is the first study to evaluate a variety of peripheral blood biomarkers before and after treatment based on the prognostic role of immunotherapy in patients with aHCC. The study is significant in the collection of an extensive range of indicators before and after treatment and in the analysis undertaken on them. Independent prognostic factors related to PFS and OS were obtained, and nomogram models were established with good results. The important clinical significance of the current study is that the required indicators are easy to calculate and can be repeated in almost all institutions without additional expenditure.

However, this is a retrospective study and some limitations must be taken into account in interpreting the conclusions. First, this is a retrospective single-center study with a limited number of patients. Future prospective studies are needed to further validate our findings. Secondly, compared with the previously reported response rate of anti-PD-1 antibody treatment in aHCC, the response rate in our study was relatively high. This may be related to a small number of study populations and the retrospective nature of our study that may lead to selection bias. Finally, non-single PD-1 inhibitors were received throughout the treatment, which inevitably caused bias.

## Conclusions

In conclusion, our study indicated that the treatment regimen, Hb at 6-week and NLR at 6-week were independent prognostic indicators for PFS, while the AFP, PLR, Hb at 6-week and NLR at 6-week were independent prognostic indicators for OS. The nomogram model based on the above peripheral blood biomarkers is helpful for us to screen the potential patients who can benefit from immunotherapy.

## Data Availability

The data that support the findings of this study are available from the Chinese People’s Liberation Army General Hospital, but restrictions apply to the availability of these data, which were used under license for the current study, and so are not publicly available. Data are however available from the author (Email: 1,610,982@mai.nankai.edu.cn) upon reasonable request and with permission of the Chinese People’s Liberation Army General Hospital.

## Abbreviations

aHCC: advanced hepatocellular carcinoma
HBV: hepatitis B virus
OS: overall survival
ICI: Immune checkpoint inhibitor
FDA: Food and Drug Administration
TMB: Tumor mu tational burden
MMR: Mismatch repair protein
MSI: Microsatellite instability
PFS: Progression-free survival
BMI: Body mass index
ECOG PS: Eastern Cooperative Oncology Group performance status
BCLC: Barcelona Clinic Liver Cancer
AFP: Alpha fetoprotein
Hb: Hemoglobin
NLR: Neutrophil-to-lymphocyte ratio
PLR: Platelet-to-lymphocyte ratio
LMR: Lymphocyte-to-monocyte ratio
SII: System immune inflammation index
AST/ALT: Aspartate Ratio of acid aminotransferase to alanine aminotransferase
ALB: Albumin
TBIL: Total bilirubin
LDH: Lactate dehydrogenase
ORR: Objective remission rate
CR: Complete remission
PR: Partial remission
DCR: Disease control rate
SD: Stable proportional disease
AUC: Area under the curve
HR: Hazard ratio
CI: Confidence interval
ROC: Receiver operating characteristic curve
